# Supplementary material for: Assessing the stability of polio eradication after the withdrawal of oral polio vaccine
Source: PLoS Biol. 2018 Apr 27;16(4):e2002468. doi: 10.1371/journal.pbio.2002468 (PMC5942853; doi:10.1371/journal.pbio.2002468)
Supplement: S1 Text — (PDF) [file pbio.2002468.s001.pdf]

S1 Text: Assessing the stability of polio eradication after the withdrawal of oral polio vaccine

Michael Famulare[1*], Christian Selinger[1], Kevin A. McCarthy[1], Philip A. Eckhoff[1], Guillaume Chabot-Couture[1]

1 Institute for Disease Modeling, Bellevue WA, USA. * mfamulare@idmod.org

Contents

Part A Parameter table 1

Part B Within-host model 3

Part B.1 Sources of data on shedding and oral susceptibility to infection . . . 3

Part B.2 Shedding duration after OPV challenge or WPV infection . . . 5

Part B.3 Concentration of poliovirus in stool . . . 6

Part B.4 Oral susceptibility to infection from OPV challenge . . . 6

Part B.5 Waning immunity against infection . . . 7

Part C Transmission model 8

Part C.1 Houston 1960 . . . 8

Part C.2 Louisiana 1953–1955 . . . 10

Part C.3 Uttar Pradesh and Bihar 2003–2008 . . . 10

Part C.4 Model fit . . . 11

Part A Parameter table

The values of all parameters used in the model, both from calibration and in the Results presentation, are shown in Table A.

Table A. Parameter table.

component	equation	parameter	value (range)	meaning
OPV-equivalent antibody titer	-	N_{Ab}	$(1, 2^{11})$	individual correlate of immunity
probability of shedding duration	(S1)	μ_S	$30.3\,(23.6, 38.6)$ days	Sabin median shedding duration ($N_{\mathrm{Ab}} = 1$)
		σ_S	$1.86\,(1.57, 2.27)$ days	Sabin scale parameter
		μ_{WPV}	$43.0\,(35.7, 51.7)$ days	WPV median shedding duration ($N_{\mathrm{Ab}} = 1$)
		σ_{WPV}	$1.69\,(1.21, 1.94)$ days	WPV scale parameter
		δ	$1.16\,(1.13, 1.21)$ days	median reduction per $\log_2(N_{\mathrm{Ab}})$
peak shedding vs age	(S2)	$S_{\max}$	$6.7\,(5.9, 7.5)$ CID50/g	maximum stool concentration at age 7 months
		$S_{\min}$	$4.3\,(3.5, 5.0)$ CID50/g	maximum stool concentration at older ages
		τ	$12\,(1, 45)$ months	decay time constant of peak concentration with age
peak shedding vs immunity	(S3)	k	$0.056\,(0.01, 0.079)$	shedding reduction with $\log_2(N_{\mathrm{Ab}})$
shedding concentration vs time	(S4)	η	$1.65\,(1.26, 2.09)$	location parameter
		ν	$0.17\,(0.01, 0.78)$	scale parameter
		ξ	$0.32\,(0.08, 0.71)$	time-dependent scale
dose response	(S5)	α	$0.44\,(0.29, 0.83)$	shape parameter
		γ	$0.46\,(0.42, 0.50)$	immunity-dependent shape parameter exponent
		β_{S1}	$14\,(3, 59)$ CID50	Sabin 1 scale parameter
		β_{S2}	$8\,(2, 30)$ CID50	Sabin 2 scale parameter
		β_{S3}	$18\,(5, 63)$ CID50	Sabin 3 scale parameter
		β_{WPV}	$2.3\,(0.3, 37)$ CID50	WPV scale parameter
waning immunity against infection	(S6)	λ	$0.87\,(0.73, 1.02)$	immunity decay exponenent
Houston Sabin transmission	(S1–6 & 1–3)	N_{Ab}	1	pre-challenge immunity (all subjects)
		dose	10^6	vaccine dose [CID50]
		p_{S1}	$0.79\,(0.70, 0.88)$	setting-specific mOPV1 modifier
		p_{S2}	$0.92\,(1.0, 0.85)$	setting-specific mOPV2 modifier
		p_{S3}	$0.81\,(0.71, 0.91)$	setting-specific mOPV3 modifier
		A_i	12 months	assumed age of index (index)
		A_h	48 months	assumed age of household member and close social contact
		T_{ih}	$5\,(1, 45)$ μg per day	fecal-oral dose from index to household member under 5 years of age
		D_{ih}	1 per day	interaction rate of index to household member pairs (assumed)
		T_{hs}	$5\,(1, 45)$ μg per day	fecal-oral dose from household member to close social contact (assumed)
		D_{hs}	$9\,(3, 46)$ per day	interaction rate of household member to close social contact pairs
Louisiana WPV (as in Houston unless shown)	(S1–6 & 1–3)	p_{Sx}	1	setting-specific mOPVx modifier
		$N_{\mathrm{Ab,sero(-)}}$	1	pre-exposure immunity (index case and seronegative household members)
		$N_{\mathrm{Ab,sero(+)}}$	93	pre-exposure immunity (seropositive household members)
		$T_{ih,young}$	$2.3\,(1.3, 5.3)$ μg per day	fecal-oral dose from index child to younger household member
		$T_{ih,adult}$	$1.3\,(0.8, 2)$ μg per day	fecal-oral dose from index child to adult household member
UP & Bihar WPV (as in Houston unless shown)	(S1–6 & 1–3)	p_{Sx}	1	setting-specific mOPVx modifier
		$N_{\mathrm{Ab},0\text{–}2}$	1	pre-exposure immunity (tOPV 0–2 doses)
		$N_{\mathrm{Ab},6+}$	512	pre-exposure immunity (tOPV 6+ doses)
		T_{ih}	$230\,(2, 18\,000)$ μg per day	fecal-oral dose from index to household member
Results (unless varied in figure)	(S1–6 & 1–3)	p_{Sx}	1	setting-specific mOPVx modifier
		A_i	12 months	assumed age of index (index)
		A_h	48 months	assumed age of household members and close social contacts
		D_{ih}	1 per day	interaction rate of index to household member pairs (assumed)
		D_{hs}	$9\,(3, 46)$ per day	interaction rate of household member to close social contact pairs (assumed)

Part B Within-host model

Part B.1 Sources of data on shedding and oral susceptibility to infection

Almost all relevant studies on OPV shedding, acquisition, and transmission published prior to 2012 were reviewed by Duintjer Tebbens *et al* [1]. Digitized data on shedding duration and concentration of poliovirus in stool were taken from the Supplementary Material of Behrend *et al* [2], corrected where discrepancies were noticed, and studies involving bOPV were added [3–5]. Dose response data were digitized from the cited references [6–9]. The analyses are broadly inclusive of published data, but this paper does not represent a systematic review with pre-specified exclusion criteria. Whole studies and trial arms were excluded if they reported evidence of substantial unmeasured exposure to poliovirus prior to OPV challenge [10–16] or when data across serotypes could not be disaggregated [17]. We included OPV challenge studies where subjects experienced low levels of natural exposure to WPV or OPV during the study, provided published evidence showed that most of the subjects were unaffected [7, 9, 18, 19]. A summary of all included data describing vaccination schedules, OPV challenge formulation or WPV exposure, ages, and shedding and dose reponse data, and possible natural exposure is provided in Table S2 [3–9, 18–31]. For a deeper discussion of data quality from reviewed studies, see Duintjer Tebbens *et al* [1].

Table B. OPV challenge studies included in analysis. Ages rounded to nearest month. "Live virus exposure" indicates possible uncontrolled exposure to OPV or WPV during study. More detailed information about the included and considered but excluded studies can be found in the digitized data tables available in S1 Code and Data and at famulare.github.io/cessationStability/.
* IPV administered at same time as OPV; † IPV administered alone but after prior OPV.

RI schedule	RI schedule	challenge	age at challenge	location	publication date	live virus exposure	shedding duration	shedding titer	dose response	reference
seronegative<br>natural	-	mOPV1<br>mOPV2<br>mOPV3	5 y<br>20 y	Nether-lands	1959	yes	yes	no	no	Verlinde1959 [20]
seronegative<br>IPVx2	-<br>-	mOPV1<br>mOPV2	13 m<br>11 m	UK	1961	no	yes	yes	no	Dick1961 [21]
seronegative	-	mOPV2	12 m	UK	1961	no	no	yes	yes	Dane1961 [32]
seronegative	-	mOPV1<br>tOPV	2 y	USA	1961	no	yes	no	no	Horstmann1961 [22]
unvaccinated	-	mOPV1<br>bOPV	0 m	USA	1962	no	yes	no	no	Holguin1962 [23]
unvaccinated<br>tOPVx3<br>IPVx3<br>IPVx4	-<br>7,8,9 m<br>2,3,4 m<br>2,3,4,15 m	mOPV1	6 m<br>16 m<br>6 m<br>16 m	UK	1966	yes	yes	yes	yes	Henry1966 [7]
unvaccinated	-	mOPV1<br>mOPV2<br>mOPV3	2 y	Japan	1966	no	yes	no	no	Takatsu1966 [24]
unvaccinated	-	mOPV1<br>mOPV2<br>mOPV3<br>tOPV	1 y	USA	1967	no	yes	no	no	Benyesh-Melnick1967 [25]
unvaccinated	-	mOPV1	2 m	UK	1981	no	no	no	yes	Minor1981 [8]
unvaccinated<br>tOPVx1	-<br>0 m	tOPV	0 or 2 m<br>2 m	China	1986	no	yes	no	no	Dong1986 [26]
tOPVx3<br>IPVx3	2,4,18 m	tOPV	2 y	USA	1991	yes	yes	no	yes	Onorato1991 [9]
unvaccinated<br>IPVx2	-<br>2,3 m	tOPV	2 m<br>4 m	Romania	1997	yes	yes	no	no	Ion-Nedelcu1997 [18]
unvaccinated<br>tOPVx1<br>tOPVx2	-<br>7 m<br>7,8 m	tOPV	7 m<br>8 m<br>9 m	France	1997	no	yes	no	no	Mallet1997 [27]
IPVx3	4,6,12 m	mOPV3	18 m	Finland	1999	no	yes	yes	no	Piirainen1999 [28]
seronegative<br>natural	-	mOPV1<br>mOPV3	65 y	Nether-lands	2005	yes	yes	no	no	Abbink2005 [29]
unvaccinated<br>tOPVx2<br>IPVx2	-<br>2,4 m<br>2,4 m	tOPV	2 m<br>6 m<br>6 m	USA	2005	yes	yes	no	no	Laassri2005 [19]
mOPV1x1<br>tOPVx1	0 m	mOPV1	1 m<br>2 y	Egypt	2008	no	yes	no	no	El-Sayed2008 [30]
IPVx2<br>& tOPVx2<br>IPVx3<br>& tOPVx3	2*,4*,7 m<br>2*,4*,7,13* m	tOPV	10 m<br>16 m	Israel	2008	no	yes	no	no	Swartz2008 [31]
tOPVxN<br>IPV boost	campaigns	mOPV1<br>mOPV3	1,5,10 y	India	2014	yes	no	no	yes	Jafari2014 [3]
IPVx1<br>& bOPVx2<br>IPVx2<br>& bOPVx1<br>IPVx3	2*,3,4 m<br>2*,3*,4 m<br>2*,3*,4* m	mOPV2	6 m	Chile	2015	no	yes	yes	no	O'Ryan2015 [4]
bOPVx3<br>& IPVx1<br>bOPVx3<br>& IPVx2<br>bOPVx3<br>tOPVx3	2,3,4* m<br>2,3,4*,8† m<br>2,3,4 m<br>2,3,4 m	mOPV2	5 or 9 m<br>9 m<br>5 m<br>5 m	Latin America	2016	no	yes	yes	no	Asturias2016 [5]

Part B.2 Shedding duration after OPV challenge or WPV infection

We assumed a log-normal survival distribution for the shedding duration given infection:

$$P\big(\text{shedding at } t \big| N_{\text{Ab}}; \text{infected at } t=0\big) = \frac{1}{2}\left(1 - \text{erf}\left(\frac{\ln(t) - (\ln(\mu) - \ln(\delta)\log_2(N_{\text{Ab}}))}{\sqrt{2}\ln(\sigma)}\right)\right), \qquad \text{(Eq A)}$$

where N_{Ab} is the OPV-equivalent antibody titer at $t=0$, μ is the median duration in days for immunologically-naive individuals ($N_{\text{Ab}} = 1$), δ describes the decrease in median duration with increasing immunity, and σ describes the shape of the distribution. The median durations and OPV-equivalent antibody titers shown in Fig. 2 were estimated under this model. Figure A shows the model maximum likelihood estimates (MLE) and 95% confidence intervals (CI) for the shedding duration distribution at low and high OPV-equivalent antibody-titers. An earlier version of this model was published within the supplemental software of Behrend *et al* [2] but was not described in that paper, and the model was used without derivation in references [33, 34]. Given the approximate aggregated survival distributions in Fig 1A, we estimated

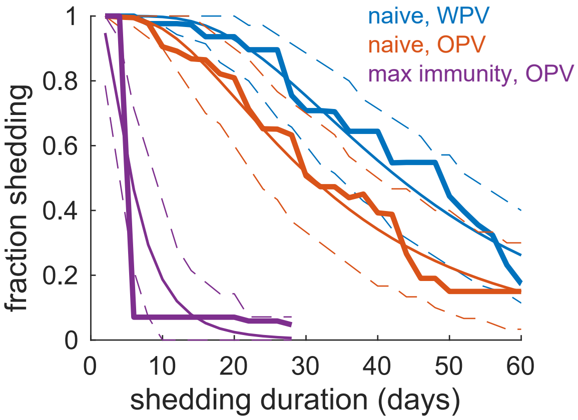

Fig A. Shedding duration probability for immunologically-naive and maximally-immune individuals. Empirical shedding duration reverse-cumulative distributions, model maximum likelihood estimate, and 95% confidence interval shown.

approximate maximum likelihood parameters of the shedding duration model using binomial maximum likelihood (assuming independent samples). We used parametric bootstrap to estimate confidence intervals.

We estimated that the WPV shedding duration in immunologically-naive children was 43.0 (35.7, 51.7) days from longitudinal surveillance studies of WPV incidence, significantly longer than our estimate for shedding duration after OPV challenge, (30.3 (23.6, 38.6) days). To confirm that this estimate is not an artifact of differences between OPV challenge and WPV surveillance study design, we examined alternative data for the time from infection to paralysis and for shedding duration after the onset of paralysis. Casey *et*

al measured that the mean time to paralysis from WPV infection is 17 days [35] and Grassly *et al* showed that the mean shedding duration after paralysis from WPV infection in UP & Bihar is 31 days [36]. The sum, 48 days, is consistent with our previous estimate.

Part B.3 Concentration of poliovirus in stool

For each trial arm that informed our concentration model [4–6, 20, 21, 28, 29], we estimated the OPV-equivalent antibody titer from the shedding duration distributions of each trial arm as above. To model the age-dependence of the concentration of poliovirus in stool, we fit an exponential model to the peak shedding concentration:

$$\log_{10}\big(\text{peak CID50/g}\big|\text{age}; N_{\text{Ab}} = 1\big) = \begin{cases} S_{\max} & \text{age} < 6 \text{ months} \\ (S_{\max} - S_{\min}) \exp\big(\frac{7-\text{age}}{\tau}\big) + S_{\min} & \text{age} \geq 6 \text{ months} \end{cases} \quad \text{(Eq B)}$$

with maximum concentration $S_{\max} = 6.7\,(5.9, 7.5)$, minumum concentration $S_{\min} = 4.3\,(3.5, 5.0)$ CID50 per gram, and time constant $\tau = 12\,(1, 45)$ months. We modeled the effect of pre-challenge immunity on concentration as:

$$\log_{10}\big(\text{peak CID50/g}\big|N_{\text{Ab}}; \text{age}\big) = (1 - k\log_2(N_{\text{Ab}}))\log_{10}\big(\text{peak CID50/g}\big|N_{\text{Ab}} = 1; \text{age}\big) \quad \text{(Eq C)}$$

with $k = 0.056\,(0.01, 0.079)$. The poliovirus concentration timeseries peaks shortly after acquiring infection and declines slowly thereafter. To model viral load over time, following refs. [2, 33], we fit a quasi-log-normal shedding profile to the age-adjusted aggregated data for immunologically-naive individuals:

$$\big(\text{concentration}(t)\big|N_{\text{Ab}}; \text{age}\big) = \max\left(10^{2.6}, \big(\text{peak CID50/g}\big|N_{\text{Ab}}; \text{age}\big)\left(\frac{\exp\Big(\eta - \frac{\nu^2}{2} - \frac{(\log(t)-\eta)^2}{2(\nu+\xi\log(t))^2}\Big)}{t}\right)\right) \quad \text{(Eq D)}$$

with $\eta = 1.65\,(1.26, 2.09)$, $\nu = 0.17\,(0.01, 0.78)$, $\xi = 0.32\,(0.08, 0.71)$, and lower bound $10^{2.6}$ CID50/g to reflect the minimum reported detectable shedding.

Part B.4 Oral susceptibility to infection from OPV challenge

For each trial arm that informed our dose response model [4–9], we estimated the OPV-equivalent antibody titer from the shedding duration distributions of each trial arm as above. In order to summarize data for all doses and OPV-equivalent antibody titers, we fit a beta-Poisson dose response model for the fraction

shedding after receiving an oral poliovirus dose. The beta-Poisson model is based on the assumptions that a single infectious unit (measured in CID50–the amount of poliovirus required to induce a cytopathic effect in 50% of inoculated cell or tissue culture plates) is sufficient to start an infection, that multiple infectious units contribute independently to the total probability of infection, and that the probability an infectious unit survives from initial oral exposure to the site of infection is beta-distributed [37]. Since the model in Behrend *et al* [2] fitted poorly at low doses and high immunity, we explored various parameterizations of the model and found that a parsimonious description of all the OPV challenge data was provided by:

$$P\big(\text{infection}\big|\text{dose}, N_{\text{Ab}}\big) = 1 - \left(1 + \frac{\text{dose}}{\beta}\right)^{-\alpha(N_{\text{Ab}})^{-\gamma}}, \qquad \text{(Eq E)}$$

where α and β are the standard beta-Poisson parameters, N_{Ab} the OPV-equivalent antibody titer, and γ captures the reduction in shedding probability with increasing immunity.

We used the fitted dose response model to estimate the OPV-equivalent antibody titer after IPV boosting on children with many prior doses of tOPV in India [3]. The maximum likelihood estimate of the OPV-equivalent antibody titer was $N_{\text{Ab}} = 3700\,(1700, 7700)$ and not significantly different from the maximal immunity produced by tOPVx3 prior to any waning [5] ($N_{\text{Ab}} = 2048\,(430, 9600)$).

Part B.5 Waning immunity against infection

For each trial arm that informed our waning model [3,5,9,20,29], we estimated the OPV-equivalent antibody titer from the shedding duration distributions of each trial arm as above.

The time interval between last immunization and mOPV challenge was either reported or estimated as follows. For individuals from tOPVx3 vaccine trials, intervals between last immunization and mOPV challenge ranged from 1 month [5] to 6 months [9]. To assess waning of tOPV-based immunity in older children, one study in Uttar Pradesh compared mOPV vaccine take rates in children 1, 5, or 10 years of age [3] who had previously recieved an unknown but high number of tOPV doses. To estimate the likely interval between last immunization and challenge, we assumed that children are offered up to 5 doses in the first year of life (3 RI plus 5 campaigns at 60% coverage), corresponding to roughly 2.5 months on average between last vaccination and mOPV challenge at 1 year of age. We assumed campaigns delivered 3 doses per year in ages two through four, corresponding to roughly 4 months between last vaccination and challenge at 5 years of age, and no doses after 5 years of age, corresponding to 5 years since last vaccination and challenge at 10 years of age. For this study, OPV-equivalent immunity was inferred via vaccine take rates using equation (5). Data on adult shedding after natural immunity were taken from studies in the Netherlands. From the

study by Verlinde *et al* [20] in 1959, the average seropositive subject in the study was 20 years of age, and we assumed that their last infection was 5 years earlier at 15 years of age when maximum seropositivity was first achieved in the population. From the study by Abbink *et al* [29] from 2005 that measured shedding in elderly individuals upon mOPV challenge, we assumed last exposure was 45 years earlier in 1960, at roughly the year in which widespread endemic transmission ceased in the Netherlands. We included data for both seropositive and seronegative adults from the Abbink *et al* study because seronegative adults showed evidence of memory immunity and reduced shedding durations in comparison to immunologically-naive children.

We fit a power law waning model [38] to the OPV-equivalent antibody titers,

$$N_{\mathrm{Ab}}(t) = \max\left(1, N_{\mathrm{Ab},1} t^{-\lambda}\right), \qquad \text{(Eq F)}$$

where t is measured in months between last immunization and oral challenge, $N_{\mathrm{Ab},1}$ is the baseline immunity one month post-immunization, and the exponent is $\lambda = 0.87\,(0.73, 1.02)$.

Part C Transmission model

Part C.1 Houston 1960

No breakdown by age was presented by Benyesh-Melnick *et al* [25] for the extrafamilial contacts of the siblings. However, because the contacts are demographically similar to the siblings and age is a significant factor for poliovirus acquisition via transmission in this setting, we used age-adjusted shedding rates in this paper. To estimate the shedding fraction in the age under 5 contact cohort, we adjusted the total reported shedding counts for each serotype as follows:

$$\text{(estimated contacts shedding under 5)} = \text{(total contacts shedding)} \times \text{(fraction siblings shedding under 5)}$$
$$\text{(estimated contacts under 5)} = \text{(total contacts)} \times \text{(fraction siblings under 5)}\,.$$

The estimated counts were rounded to the nearest integer and confidence intervals presented are based on the rounded estimated counts.

Figure B shows more information about the age-dependence of shedding after mOPV challenge. Older index children shed slightly less after mOPV challenge than younger children for types 2 and 3 (type 1 $p = 0.105$; type 2 $p = 0.016$; type 3 $p = 0.025$; two-tailed Fisher's exact test). This observation was not explored in the original paper, and we propose two possible explanations. As described in Table 1 of

Benyesh-Melnick *et al* [25], older index children were more likely to have received at least one dose of IPV. However, it should be noted that the original authors reported that they found no significant differences between IPV and unvaccinated index subjects, as is compatible with our metastudy. A second possibility is that stool concentrations of poliovirus are higher in young index cases, and so stool culture may be more sensitive to shedding in younger children (eq. (Eq B)). In the household member cohorts, there were no

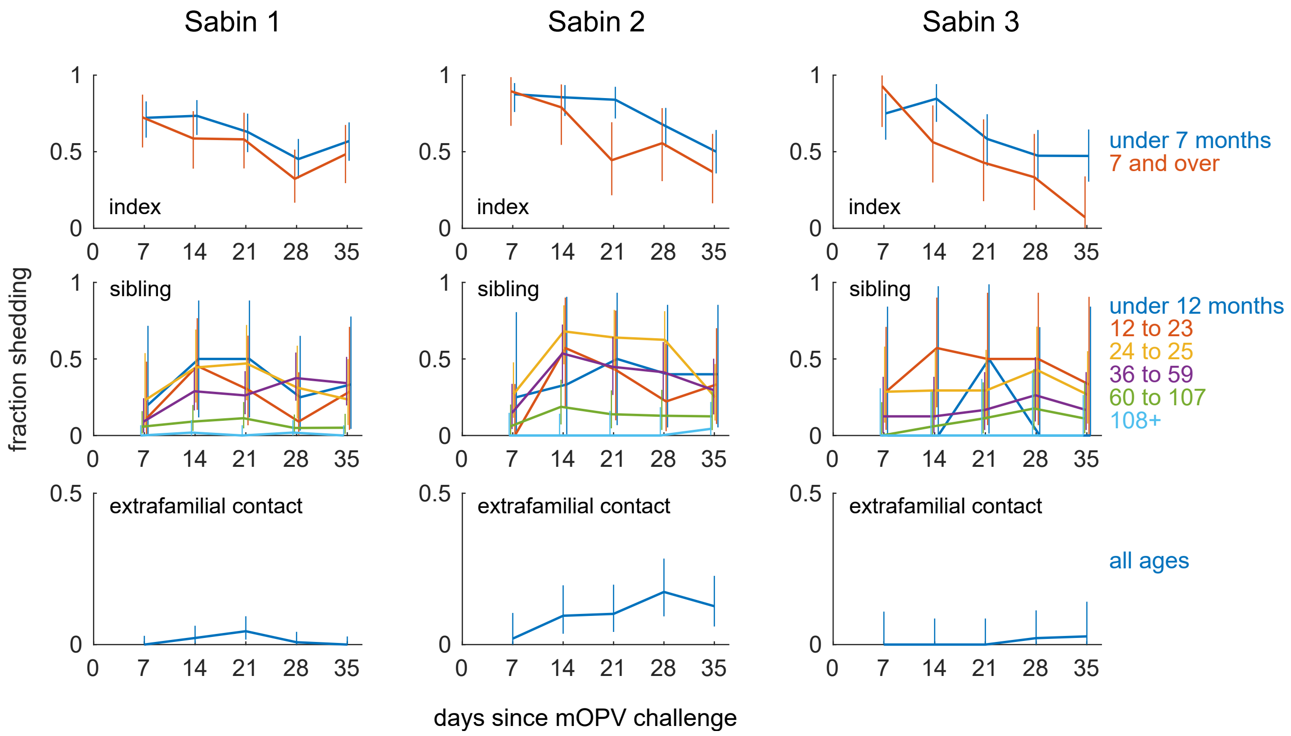

Fig B. Fraction shedding by cohort and age range as originally reported. Observed fraction shedding and estimated 95% binomial confidence interval for each serotype, subject type, and reported age cohort.

statistically significant differences in shedding among the age groups under 12 months, 12 to 23 months, 24 to 35 months, or 36 to 59 months for any serotype. However, there was significantly less shedding in the 60 to 107 month age group relative to the 36 to 59 age group ($p < 0.001$ for all serotypes). As stated in the main text, shedding in siblings age 60 to 107 months (5 to 9 years) is significantly below that of ages less than 5 years for all serotypes (type 1 $p < 0.001$; type 2 $p < 0.001$; type 3 $p = 0.002$). Shedding rates were very low in parents and children age 10 years and older ($< 2\%$) [25], and so it is likely the transmission was direct from index child to sibling and was not mediated by infected caretakers. Shedding due to transmission-acquired type 2 was significantly more common than for types 1 and 3, and shedding due to transmission was similar for types 1 and 3 (mean prevalence: type 1 vs type 2 $p = 0.002$; type 1 vs type 3 $p = 0.33$). Primary extrafamilial contacts of siblings exhibited a similar pattern of increased type 2 shedding

and comparable type 1 and 3 shedding (type 1 vs type 2 $p < 0.001$; type 1 vs type 3 $p = 0.73$). Although the authors did not describe the relationships between siblings and extrafamilial contacts in detail, it is likely that the contacts were close friends of the siblings and were directly infected by the siblings, as the authors also describe a smaller set of more socially-distant "secondary extrafamilial contacts" who "were drawn from the neighborhoods or schools attended by the siblings" and who were infected at lower rates than the primary contacts [25].

Little information about shedding in secondary contacts was provided, except to note that, summed across all trial arms, 15 of 280 secondary contacts were positive for Sabin 2 and the highest incidence rate was 13% in the secondary extra-familial contacts of tOPV recipients. Assuming the number of secondary contacts is proportional to the number of primary contacts for each trial arm, $n = 10$ of the type 2 positives were in secondary contacts of tOPV recipients (13% of trial arm total) and $n = 5$ were in secondary contacts of mOPV2 recipients (8.5% of trial arm total).

Part C.2 Louisiana 1953–1955

We calibrated model incidence to the seroconversion data reported in Gelfand *et al* [39]. Stool collection data was also available, but it reported lower levels of incidence. This was likely due to missing infections: the average interval between samples was 27 days while the average shedding duration in seropositive subjects with median $N_{\text{Ab}} = 93$ is only 16 days under our model. We assumed 100% incidence of immunologically-naive index children after WPV exposure, based on the study design that reported household member incidence conditional on detection of the child's first natural infection with poliovirus.

Part C.3 Uttar Pradesh and Bihar 2003–2008

We calibrated our model to the estimates of mean stool prevalence after the onset of paralysis in close contacts of WPV cases reported by Grassly *et al* [36]. We assumed that the contact data best corresponded to household members in our model. Quote:

> *During identification of healthy contacts, an effort was made to include those children with the closest contact to the individual with suspected poliomyelitis, such as siblings, playmates, or residents of the same household.* [36]

To shift our model from prevalence after infection to prevalence after paralysis, we convolved our prevalence timeseries with the time-to-paralysis distribution:

$$P_{\text{family}}\big(\text{shedding at } t \big| \text{index paralysis at } t=0\big) = \int_0^t dt' P_{\text{family}}(\text{shedding at } t')\, p_{\text{paralysis}}(t-t')\,, \qquad \text{(Eq G)}$$

where $p_{\text{paralysis}}(t)$ was given by the histogram in Figure 2 of Casey *et al* [35]; the mean time from infection to the onset of paralysis was 17 days. The model was calibrated against the mean of eq. (Eq G) over the first 90 days after index child paralysis.

Part C.4 Model fit

Figure C is an extension of Figure 6 that shows maximum likelihood estimates of the fraction shedding (prevalence) and cumulative incidence in our model for the three calibration targets. For parameters, see Table A.

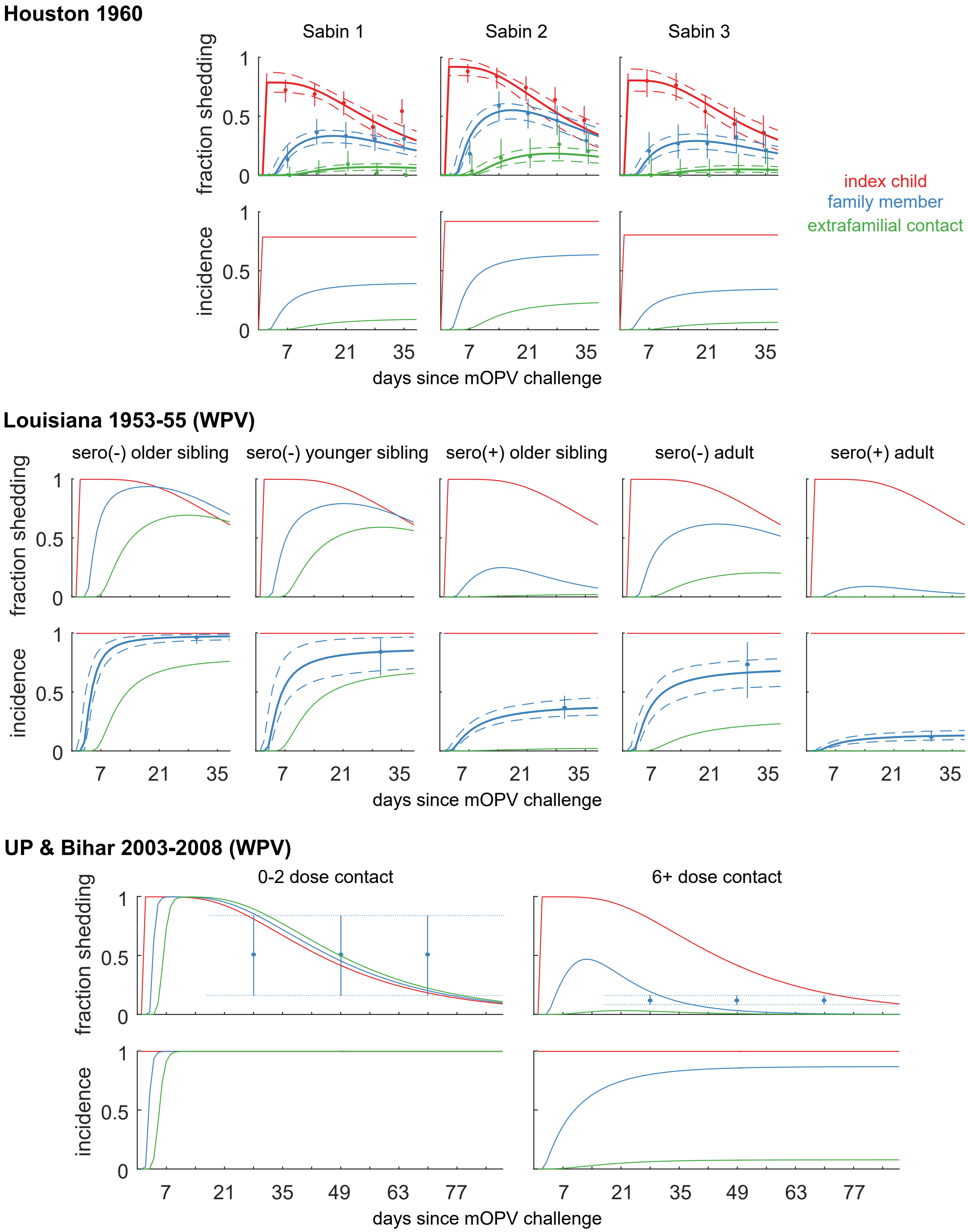

Fig C. Model of fraction shedding and incidence for each calibration target. Maximum likelihood estimates (solid lines) are shown for each subject type (color) after mOPV in Houston or WPV in Lousiana and UP & Bihar. Dots-and-whiskers show calibration targets, and model 95% CI are shown for comparison. For Houston, we compared fraction shedding in stool to model prevalence. For Louisiana, cumulative incidence one month after index child infection. For UP & Bihar, we compared the mean fraction of close (direct personal) contacts shedding after index child paralysis (model prevalence and calibration target (mean over time) shown).

References

1. Duintjer Tebbens RJ, Pallansch MA, Kim JH, Burns CC, Kew OM, Oberste MS, et al. Oral poliovirus vaccine evolution and insights relevant to modeling the risks of circulating vaccine-derived polioviruses (cVDPVs). Risk analysis. 2013;33(4):680–702. doi:10.1111/risa.12022.

2. Behrend MR, Hu H, Nigmatulina KR, Eckhoff P. International Journal of Infectious Diseases A quantitative survey of the literature on poliovirus infection and immunity. International Journal of Infectious Diseases. 2013; p. 1–10. doi:10.1016/j.ijid.2013.09.005.

3. Jafari H, Deshpande JM, Sutter RW, Bahl S, Verma H, Ahmad M, et al. Polio eradication. Efficacy of inactivated poliovirus vaccine in India. Science (New York, NY). 2014;345(6199):922–5. doi:10.1126/science.1255006.

4. O'Ryan M, Bandyopadhyay AS, Villena R, Espinoza M, Novoa J, Weldon WC, et al. Inactivated poliovirus vaccine given alone or in a sequential schedule with bivalent oral poliovirus vaccine in Chilean infants: a randomised, controlled, open-label, phase 4, non-inferiority study. The Lancet Infectious Diseases. 2015;3099(15). doi:10.1016/S1473-3099(15)00219-4.

5. Asturias EJ, Bandyopadhyay AS, Self S, Rivera L, Saez-llorens X, Lopez E, et al. Humoral and intestinal immunity induced by new schedules of bivalent oral poliovirus vaccine and one or two doses of inactivated poliovirus vaccine in Latin American infants: an open-label randomised controlled trial. Lancet. 2016;6736(16):1–12. doi:10.1016/S0140-6736(16)00703-0.

6. Dane DS, Dick GW, Briggs M, Nelson R, McAlister J, Connolly JH, et al. Vaccination against poliomyelitis with live virus vaccine: 8. Changes in Sabin Type 1 oral vaccine virus after multiplication in the intestinal tract. British medical journal. 1961; p. 269–271.

7. Henry JL, Jaikaran ES, Davies JR, Tomlinson AJ, Mason PJ, Barnes JM, et al. A study of poliovaccination in infancy: excretion following challenge with live virus by children given killed or living poliovaccine. The Journal of hygiene. 1966;64(1):105–20.

8. Minor TE, Allen CI, Tsiatis AA, Nelson DB, D'Alessio DJ. Human infective dose determinations for oral poliovirus type 1 vaccine in infants. Journal of clinical microbiology. 1981;13(2):388–9.

9. Onorato IM, Modlin JF, McBean aM, Thoms ML, Losonsky Ga, Bernier RH. Mucosal immunity induced by enhance-potency inactivated and oral polio vaccines. The Journal of infectious diseases. 1991;163(1):1–6.

10. Gelfand HM, Potash L, Leblanc DR, Fox JP. Intrafamilial and Interfamilial Spread of Living Vaccine Strains of Polioviruses. Journal of the American Medical Association. 1959;.

11. Sabin AB, Ramos-Alvarez M, Alvarez-Amezquita J, Pelon W, Michaels RH, Spigland I, et al. Live, orally given poliovirus vaccine: effects of rapid mass immunization on population under conditions of massive enteric infections with other viruses. Journal of the American Medical Association. 1960;173:1521–1526. doi:10.1007/BF02721844.

12. Domok I, Molnar E, Jancso a. Virus excretion after mass vaccination with attenuated polioviruses in Hungary. British medical journal. 1961;1(5237):1410–7.

13. Goffe A, Pollock TM, Shand FL. Vaccination of adults with a British oral poliomyelitis vaccine prepared from Sabin strains. British medical journal. 1961;2(5247):272–274.

14. Kogon A, Spigland I, Elveback L, Fox JP. Oral poliovirus vaccination in middle income families. American journal of epidemiology. 1965;82(1):14–26.

15. Glezen WP, McCollough RH, Lamb GA, Chin TDY. Quantitative relationship of preexisting homotypic antibodies to excretion of poliovirus types 1, 2 and 3 following the feeding of trivalent attenuated poliovirus vaccine. American Journal of Epidemiology. 1969;90(2):146–156.

16. Samoilovich EO, Feldman EV, Yermalovich MA, Protas II, Titov LP. Vaccine-associated paralytic poliomyelitis and other diseases with acute flaccid paralysis syndrome in Belarus. Central European journal of public health. 2003;11(4):213–8.

17. Maldonado Ya, Peña-Cruz V, de la Luz Sanchez M, Logan L, Blandón S, Cantwell MF, et al. Host and viral factors affecting the decreased immunogenicity of Sabin type 3 vaccine after administration of trivalent oral polio vaccine to rural Mayan children. The Journal of infectious diseases. 1997;175(3):545–53.

18. Ion-Nedelcu N, Strebel PM, Toma F, Biberi-Moroeanu S, Combiescu M, Persu A, et al. Sequential and Combined Use of Inactivated and Oral Poliovirus Vaccines: Dolj District, Romania, 1992-1994. Journal of Infectious Diseases. 1997;175(Suppl1):S241–6.

19. Laassri M, Lottenbach K, Belshe R, Wolff M, Rennels M, Plotkin S, et al. Effect of different vaccination schedules on excretion of oral poliovirus vaccine strains. The Journal of infectious diseases. 2005;192(12):2092–8. doi:10.1086/498172.

20. Verlinde JD, Wilterdink JB. A small-scale trial on vaccination and re-vaccination with live attenuated polioviruses in the Netherlands. In: Preliminary Report on Mass Oral Immunization of Population Against Poliomyelitis with Live Virus from A.B. Sabin's Attenuated Strains. World Health Organization; 1959. p. 355–367.

21. Dick GW, Dane DS, McAlister J, Briggs M, Nelson R, Field CMB. Vaccination against poliomyelitis with live virus vaccines. 7. Effect of previous Salk vaccination on virus excretion. British medical journal. 1961;2(5247):266–269.

22. Horstmann DM, Paul JR, Godenne-McCrea M, Green RH, Opton EM, Holtz AI, et al. Immunization of Preschool Children with Oral Poliovirus Vaccine (Sabin). JAMA: The Journal of the American Medical Association. 1961;178(7):693–701.

23. Holguin AH, Reeves JS, Gelfand HM. Immunization of infants with the Sabin oral poliovirus vaccine. American Journal of Public Health. 1962;52(4).

24. Vaccine Administration Subcommittee. Evaluation of Sabin live poliovirus vaccine in Japan: II. Clinical, virologic, and immunologic effects of vaccine in children. Japanese Journal of Medical Science and Biology. 1966;19:277–291.

25. Benyesh-Melnick M, Melnick JL, Rawls WE, Wimberly I, Oro JB, Ben-Porath E, et al. Studies of the immunogenicity, communicability and genetic stability of oral poliovaccine administered during the winter. American journal of epidemiology. 1967;86(1):112–36.

26. Dong DX, Hu XM, Liu WJ, Li JS, Jin YC, Tan SG, et al. Immunization of neonates with trivalent oral poliomyelitis vaccine (Sabin). Bulletin of the World Health Organization. 1986;64(6):853–60.

27. Mallet L, Pelloquin F, Brigaud M, Caudrelier P, Bandet R, Xueref C, et al. Comparative study of poliovirus excretion after vaccination of infants with two oral polio vaccines prepared on vero cells or on primary monkey kidney cells. Journal of Medical Virology. 1997;52(1):50–60. doi:10.1002/(SICI)1096-9071(199705)52:1¡50::AID-JMV9¿3.0.CO;2-Q.

28. Piirainen L, Stenvik M, Roivainen M, Eskola J, Beuvery EC, Hovi T. Randomised, controlled trial with the trypsin-modified inactivated poliovirus vaccine: assessment of intestinal immunity with live challenge virus. Vaccine. 1999;17(9-10):1084–90.

29. Abbink F, Buisman AM, Doornbos G, Woldman J, Kimman TG, Conyn-van Spaendonck MaE. Poliovirus-specific memory immunity in seronegative elderly people does not protect against virus excretion. The Journal of infectious diseases. 2005;191(6):990–9. doi:10.1086/427810.

30. El-Sayed N, Yehia EG, Abbassy AA, Seoud I, Salama M, Kandeel A, et al. Monovalent type 1 oral poliovirus vaccine in newborns. New England Journal of Medicine. 2008;359(16):1655–1665.

31. Swartz TA, Green MS, Handscher R, Sofer D, Cohen-Dar M, Shohat T, et al. Intestinal immunity following a combined enhanced inactivated polio vaccine/oral polio vaccine programme in Israel. Vaccine. 2008;26(8):1083–90. doi:10.1016/j.vaccine.2007.12.021.

32. Dane DS, Dick GW, Briggs M, Nelson R, McAlister J, Connolly JH, et al. Vaccination Against Poliomyelitis with Live Virus Vaccines: 6. Changes in Sabin Type II Oral Vaccine Virus After Human Passage. British medical journal. 1961;2(5247):259–265.

33. Wagner BG, Behrend MR, Klein DJ, Upfill-Brown AM, Eckhoff PA, Hu H. Quantifying the impact of expanded age group campaigns for polio eradication. PloS one. 2014;9(12):e113538. doi:10.1371/journal.pone.0113538.

34. Famulare M, Chang S, Iber J, Zhao K, Adeniji JA, Bukbuk D, et al. Sabin vaccine reversion in the field: a comprehensive analysis of Sabin-like poliovirus isolates in Nigeria. Journal of Virology. 2016;90(1):317–331. doi:10.1128/JVI.01532-15.

35. Casey AE. The incubation period in epidemic poliomyelitis. Journal of the American Medical Association. 1942;120(11):805–807.

36. Grassly NC, Jafari H, Bahl S, Durrani S, Wenger J, Sutter RW, et al. Asymptomatic wild-type poliovirus infection in India among children with previous oral poliovirus vaccination. The Journal of infectious diseases. 2010;201(10):1535–43. doi:10.1086/651952.

37. Furumoto WA, Mickey R. A mathematical model for the infectivity-dilution curve of tobacco mosaic virus: Theoretical considerations. Virology. 1967;32(2):216–223. doi:10.1016/0042-6822(67)90271-1.

38. Teunis PFM, van Eijkeren JCH, de Graaf WF, Marinović AB, Kretzschmar MEE. Linking the seroresponse to infection to within-host heterogeneity in antibody production. Epidemics. 2016;16:33–39. doi:10.1016/j.epidem.2016.04.001.

39. Gelfand HM, Leblanc DR, Fox JP, Conwell DP. Studies on the development of natural immunity to poliomyelitis in Louisiana. II. Description and Analysis of Episodes of infection observed in study group households. American Journal of Hygeine. 1957;65:367–385.
